# Food Composition and Dedicated Databases: Key Tools for Human Health and Public Nutrition (2nd Edition)

**DOI:** 10.3390/nu17071119

**Published:** 2025-03-24

**Authors:** Alessandra Durazzo, Massimo Lucarini

**Affiliations:** CREA-Research Centre for Food and Nutrition, Via Ardeatina 546, 00178 Rome, Italy

## Introduction

A detailed knowledge of the presence and level of components of food is documented in food composition databases and represents a key factor to study the relationship between diet and health. Food composition and dedicated databases represent resources and tools for stakeholders in many fields, supporting human research studies, evidence-based policymaking, and consumer education.

Detailed documentation of all information concerning foods, components, values, and references is a key element in maintaining a food composition database, collecting and integrating data from different sources and also taking into account different aspects such as biodiversity, climate change, consumer preferences, and global market trade.

The standardization, harmonization, and integration of food data sources represent a challenge throughout worldwide in the era of big data in the perspective of open science and transdisciplinary research.

Global harmonization in methodology is needed among databases to allow data interchange. Ontology alignment is crucial for data integration and interoperability across multiple applications across diverse disciplines.

In recent decades, significant advancements have been made in the development of advanced methods and systems for ontology alignment, also now taking into account the utilization of artificial intelligence in infrastructure systems and ontology and coding systems.

The Special Issue “Food Composition and Dedicated Databases: Key Tools for Human Health and Public Nutrition (2nd Edition)” aimed at covering all aspects linked to food composition and dedicated databases. The overall goal is given by a collection of main features and roles, advances, and applications of databases as tools for human health and public nutrition on a global scale.

The reliable use of food composition and dedicated databases in the current documented initiatives is reported.

The use and examples of new and automatized technologies in management data are described. 

The standardization, harmonization and FAIRization of data for organizing and exploiting food data into various applications is new frontier, particularly linking resources, information and data for food, environmental, nutrition and health, throughout an integrating modelling. 

Yeung et al. [1] carried out a bibliometric analysis of Food Composition Databases (FCDBs) by identifying the most productive authors, institutions, and journals concerning this matter.

Wakayama et al. [2] aimed to classify foods based on nutrient information in the Standard Tables of Food Composition in Japan, 2020 (Eighth Revised Edition) by using a novel technique—t-distributed stochastic neighbor embedding—to visualize high-dimensional data; the authors [2] marked how the visualization of food composition could enable an enhanced comprehensive understanding of the nutrients in foods, which could lead to novel aspects of nutrient-value-based food classifications.

Durazzo et al. [3] developed a database of LanguaL^TM^ and FoodEx2 codes of 50 food preparations and ready-to-eat dishes designed for consumption outside the home; it represents a tool for data standardization and data sharing in several potential applications, such as nutritional cards, nutritional facts, and food labels, or booklets and brochures, for the promotion of food products [3].

The study of Steenbergen et al. [4] is addressed to the Nutri-Score in the European food retail supply.

Federici et al. [5] described the “Healthy Snack” project, a health promotion project focused on correct nutrition in childhood, aimed at evaluating changes in the choice of mid-morning snacks made at school, following nutritional education interventions implemented in the 2022–2023 school year by the Prevention Department of ULSS 1 Dolomiti at primary schools in the Belluno area that joined the project. The results collected in the annual survey period were related to the type and quantity of snacks consumed at school, and allowed students to gain a final score, comparing the period before and after the educational intervention to demonstrate the effectiveness of the actions promoted by healthcare workers and the increased nutritional quality of meals [5]. The authors [5] also marked how intervening in the school context, where most children spend a significant part of the day, is advantageous, and can have a significant impact on eating behaviors that can be learned and maintained over time, contributing to a reduced prevalence of overweight and obesity, and consequently preventing cardiometabolic pathologies throughout life [5].

Ficco et al. [6] described the state of the art of available information and its potential use in epidemiological studies for polyphenols in cereals.
